# *Sox9* regulates cell state and activity of embryonic mouse mammary progenitor cells

**DOI:** 10.1038/s42003-018-0215-3

**Published:** 2018-12-13

**Authors:** Naoko Kogata, Philip Bland, Mandy Tsang, Erik Oliemuller, Anne Lowe, Beatrice A. Howard

**Affiliations:** 1 The Breast Cancer Now Toby Robins Research Centre, Division of Breast Cancer Research, The Institute of Cancer Research, London, UK; 2 Present Address: Cellular Signalling and Cytoskeletal Function Lab, The Francis Crick Institute, London, UK

**Keywords:** Stem cells, Organogenesis

## Abstract

Embryonic mammary cells are a unique population comprised of undifferentiated, highly plastic progenitor cells that create normal mammary tissues. The mammary gland continues to develop after birth from descendants of embryonic mammary cells. Here, we establish cell lines from mouse mammary organs, immediately after they formed during prenatal development, to facilitate studies of primitive mammary cells, which are difficult to isolate in sufficient quantities for use in functional experiments. We show that some lines can be induced to secrete milk, a distinguishing feature of mammary epithelial cells. Targeted deletion of *Sox9*, from one clone, decreases the ability to respond to lactogenic stimuli, consistent with a previously identified role for *Sox9* in regulating luminal progenitor function. *Sox9* ablation also leads to alterations in 3D morphology and downregulation of Zeb1, a key epithelial–mesenchymal transition regulator. Prenatal mammary cell lines are an invaluable resource to study regulation of mammary progenitor cell biology and development.

## Abstract (web summary)

Naoko Kogata et al. generated murine mammary progenitor cell lines that form spheres and secrete milk upon hormonal stimulation. Deletion of *Sox9* increased the ability of these cells to forms spheres but decreased milk production induced by lactogenic stimuli, consistent with the role of this transcription factor on maintaining the stem cell state.

## Introduction

Embryonic breast epithelial cells are a unique cell population comprised of undifferentiated and highly plastic progenitor cells that ultimately give rise to all other postnatal breast epithelial cells. Lineage tracing studies have indicated that embryonic mammary cells are multipotent in vivo^1–3^. An important area of research in mammary gland biology is to determine the roles of genes and signalling pathways that regulate embryonic stages of mammary gland development, as many of these are also relevant to processes that are deregulated in cancer^4,5^. Despite their relevance to breast cancer research, the routine use of primary mid-gestation embryonic mammary cells for functional study is not currently feasible, due to the small size of the nascent organ.

In mice, mammary gland development commences at embryonic day 11 (E11) with the sequential appearance of five pairs of mammary primordia^6^. Local epithelial thickenings invaginate to the underlying tissue to form buds, which from E12.5 onwards are surrounded by a specialised condensed mammary mesenchyme (MM). Mammary buds grow relatively slowly in size until E14 when the epithelial cells within the bud start to proliferate extensively and then invade into the underlying mesenchymal tissues^6^. These early stages of development are of particular interest as the cells have a number of unique properties. The epithelial cells within the E11–E13-stage mammary organ are largely quiescent^7,8^. At these stages of development, epithelial cells are thought to accrue within the mammary organ via localised cell movements^9,10^. Dissociated embryonic mammary cells from E12- to E13-stage organs have minimal ability to efficiently repopulate cleared mammary fat pads, whilst cells from E160- toE18-stage organs have a much higher ability to engraft^11,12^. Intact mammary bud epithelium from E13-stage embryos can repopulate cleared fat pads, suggesting that a stem cell population has been delimited by mid-gestation^13^. Recent results from lineage tracing experiments indicate that embryonic mammary cells at E12–E13 stages are multipotent at the cellular level and become lineage restricted prior to birth^1–3^. Although embryonic mammary progenitor cells from E12 to E13 stages harbour very distinct biological properties from embryonic mammary progenitor cells isolated from E16 to E17 stages and from the postnatal mammary gland, a lack of appropriate in vitro models has limited their accessibility for many researchers.

Most studies of the embryonic mammary gland have relied on analyses of embryos from genetically modified mice or embryonic mammary organ explant cultures^14–17^. These methods require considerable training, expertise and the use of animals. However, using these novel embryonic mammary cell lines and standard two- and three-dimensional (2D and 3D) culture techniques, we model several key aspects of embryonic mammary gland development in vitro. Using CRISPR-Cas9 genome editing, we investigate the role of Sox9, an embryonic Sox transcription factor, which has been implicated in conferring stem cell state to differentiated postnatal mammary epithelial cells (MECs)^18^, in the regulation of stem cell activity and the differentiation potential of cells formed during early stages of embryonic mammary gland development. Our findings highlight the distinct biological features and context-dependent regulation of embryonic mammary progenitor cells and are a novel resource for studying this unique cell population.

## Results

### Establishment of embryonic mammary progenitor cell lines

**Fig. 1:**
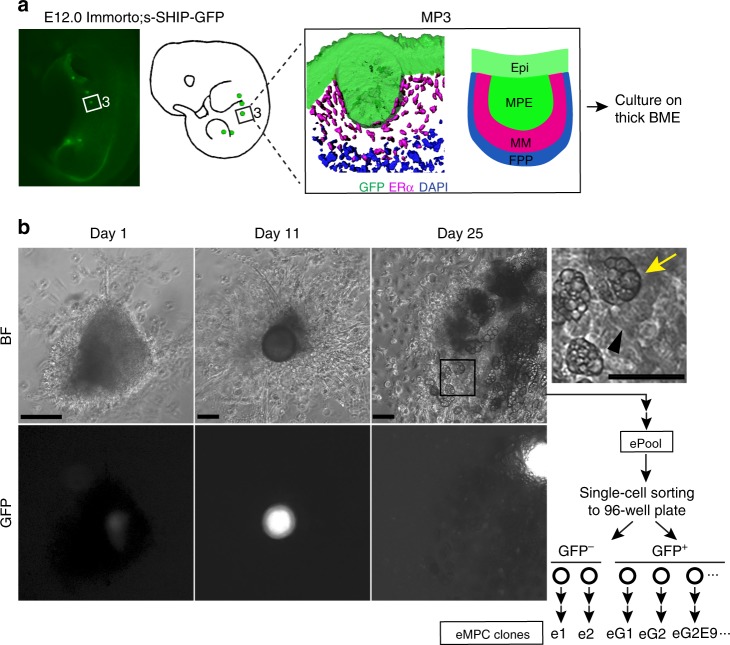
Establishment of embryonic mammary progenitor cell lines. **a** Schematic illustration of experimental scheme of eMPC derivation from E12.0-stage Immorto;s-SHIP-GFP mammary organ number three. After microdissection of MP3 to include three tissues: GFP+ MPE (GFP staining in green), GFP−, ERα+ MM (ERα staining in magenta) and adjacent ERα− FPP (DAPI staining in blue), MP3 was cultured on thick BME for 4 weeks. **b** Bright-field and GFP images of cultured MP3 at day 1, day 11 and day 25. After culture, a variety of cell types including lipid-containing adipocyte-like cells (area shown in black box and yellow arrow), as well as contractile muscle-like cells (black arrowhead) were observed within the cell population. After enzymatic dissociation, eMPCs were plated and expanded in 2D culture to obtain a pool of eMPCs (ePool). eMPC cells were subject to single-cell sorting into GFP+ and GFP− cell populations using fluorescence-activated cell sorting. Clones were expanded as single-cell-derived clones to create the 18 single-cell-derived clones from GFP− cells (e1 and e2) or GFP+ cells (eG1, eG2E9, etc.) described in this study. Scale bar, 200 μm. MP3, mammary primordium 3; MPE, mammary primordial epithelium; Epi, epithelium; MM, mammary mesenchyme; FPP, fat pad precursor; eMPCs, embryonic mammary progenitor cells; BF, bright field

To date, the establishment and maintenance of primary mammary embryonic epithelial cell cultures from mouse embryos at stages between E12 and E13 has not been possible. To overcome this issue, we have taken advantage of the Immortomouse, a genetically modified mouse, to introduce a temperature-sensitive Simian virus (SV) 40 antigen under control of an interferon (IFN)-regulated promoter that enables immortalisation of certain types of cells, including epithelial and mesenchymal cells^19^. Immortomice were bred with s-SHIP-GFP mice, which green fluorescent protein (GFP) expression marks epithelial progenitor cells, including those in the embryonic mammary primordia to facilitate and confirm dissection of the mammary organ when it is first morphologically distinct^20^. The majority of s-SHIP-GFP embryonic MECs are GFP+ and adjacent embryonic mammary mesenchymal cells are GFP− (Fig. 1a and Supplementary Fig. 1A). Mammary primordia number three were micro-dissected from E12.0-stage embryos so that the mammary epithelium, MM and fat pad precursors (FPPs) were isolated and such that other GFP+ cell types marked in s-SHIP-GFP embryos^21^ were excluded (Fig. 1a). Excised mammary organs were embedded in thick basement membrane extract (BME) for a month, so that embryonic mammary progenitor cells (eMPC) from the mammary organoid were able to proliferate (Fig. 1b). This adaptation period appeared to be important for successful cell isolation since enzymatic digestion of Immorto:s-SHIP-GFP mammary organs immediately after microdissection did not give rise to cultivable eMPCs beyond 1 month. After proliferating for 1 month, eMPCs were harvested by enzymatically digesting the organoid culture grown in thick BME and expanding the cells in 2D culture. The bulk cells of this expanded eMPC are referred to subsequently as ePool (Fig. 1b). To obtain clonal cell lines, single ePool cells were separated using fluorescence-activated cell sorter according to s-SHIP-GFP expression status (GFP+ or GFP−) at the time of sorting (Fig. 1b and Supplementary Fig. 1B). Approximately 9% of GFP+ and ~2% of GFP− cells gave rise to viable clones. Sixteen clones were derived from the GFP+ fraction (designated eG1, eG2, eG2E9, etc). e1 and e2 were the only clones that survived from GFP− fraction (Supplementary Fig. 1C). However, GFP expression is not necessarily indicative of tissue of origin since the activity of s-SHIP promoter is variable in cell populations expanded in 2D culture. eMPC clones expanded from single s-SHIP-GFP+ or single s-SHIP-GFP− cells contain both GFP+ and GFP− cells upon passage in 2D culture (Supplementary Fig. 1C).

**Fig. 2:**
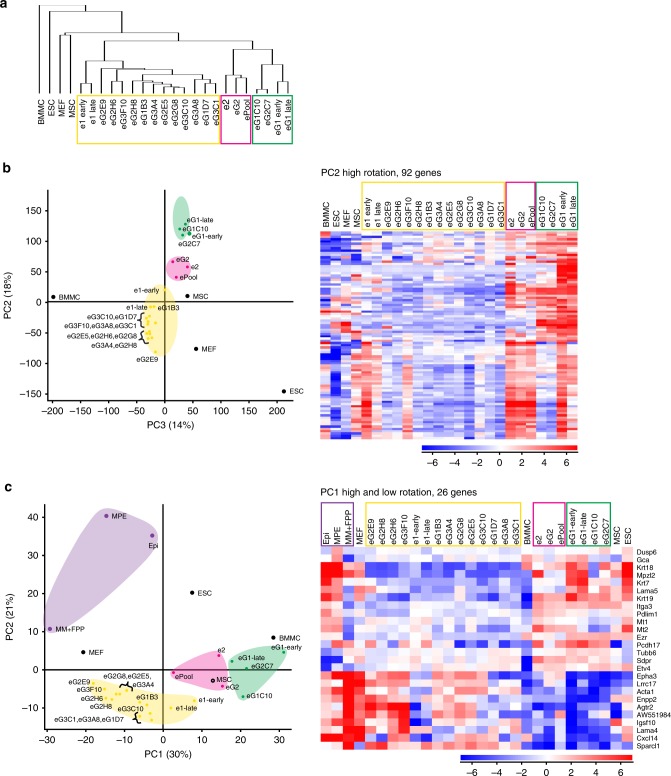
Global gene expression landscape of embryonic mammary progenitor cell lines. **a** Dendrograms from hierarchical clustering of RNA-sequencing data using 2059 most significantly changing genes of eMPC and other types of progenitor/stem cells. Eighteen eMPC clones are classified into three main clusters, which are highlighted: e1 cluster (yellow box), e2/eG2/ePool cluster (magenta box) and eG1 cluster (green box). **b** PCA of eMPC and other types of progenitor/stem cells. Loading plot using principal component (PC) 2 (18%) and PC3 (14%) of the 17,204 most variable genes. Three main eMPC clusters (yellow, magenta and green) recapitulate the hierarchical clustering results shown in **b**. The heatmap shows hierarchical clustering based on top 0.5% of PC2 (92 genes). **c** PC1 and PC2 loading plot using the most highly significant genes in the dataset of eMPC and other progenitor/stem cells combined with embryonic mammary tissues. Three eMPC clusters (yellow, magenta and green) and an embryonic mammary tissue cluster (purple) from PCA are highlighted based on PC1. The heatmap shows results of hierarchical clustering based on 26 genes from PC1 (top high and low rotation genes analysed using the likelihood ratio test (LRT) as well as the intensity difference test). eMPC, embryonic mammary progenitor cell; MP3, mammary primordium 3; BME, basement membrane extract; PC, principal component; MPE, mammary primordial epithelium; Epi, epithelium; MM, mammary mesenchyme; FPP, future fat pad precursor; ESC, embryonic stem cells; MEF, mouse embryonic fibroblasts; MSC, mesenchymal stem cells; BMMC, bone marrow mononuclear cells. Early and late indicate clones that were sequenced at early (5th) and later (13th) passage

Hierarchical cluster analysis of gene expression profiles obtained by RNA-sequencing shows that eMPCs are distinct from other types of progenitor and stem cells, including embryonic stem cells (ESCs), mouse embryonic fibroblasts (MEFs), mesenchymal stem cells (MSCs) and bone marrow mononuclear cells (BMMCs) (Fig. 2a). From 17,204 significantly modulated genes, the 2059 most highly variable were selected when the DESeq2 software was employed with the likelihood ratio test (LRT), and using the intensity difference test (detailed selection strategy and gene lists are in Supplementary Data 1). Principal component analysis (PCA), based on 92 genes, produces a similar clustering of the eMPC clones with other stem cell types (Fig. 2b and Supplementary Data 1).

Mammary organs from C57BL/6 E12.5-stage mouse embryos were micro-dissected so that mammary epithelium, mammary stroma (MM, and adjacent FPP) and surface epithelium were separated and the tissues were used for gene expressing profiling using RNA-sequencing. Hierarchical clustering based on transcriptome profiles of the separated embryonic mammary primordia tissues and eMPCs and other stem cell types was performed (Supplementary Fig. 2 and Supplementary Data 2). To assess the global landscape of expression in this dataset, we performed PCA and 26 genes revealed clustering of eMPC lines into three distinct groups based on their expression of genes encoding keratin and cell adhesion regulators (Fig. 2c). Our previous results have shown that embryonic mammary cells are largely devoid of cells expressing differentiation markers, although a few cells present within the E12-stage embryonic mammary bud epithelium express stem cell/progenitor markers associated with mature postnatal MEC lineages^22^. PCA identifies clusters based on expression of several genes, some of which control cell adhesion, including the protocadherin, *Pcdh7*, integrin, *Itga3*, and extracellular matrix (ECM) ligand, *Lama4*, that are highly expressed by a subset of the cell lines (eG1, eG2C7, eG1C10), which also express keratins (*Krt7*, *Krt18*, *Krt19*). These clones belonging to the cluster marked in green in Fig. 2 may represent mammary epithelial progenitor cells. Clones belonging to another cluster, marked in yellow in Fig. 2, including e1, which was selected further study, express high levels of a number of markers including, *Cd34*, *Cd74, Ebf2, Fabp4*, *Runx2*, and *Sox9* that are associated with a variety of stem cell types^23–28^ and may represent less-differentiated progenitor/stem cells types. Two cells lines with less distinct features (marked in orange in Fig. 2), e2 and eG2 that cluster with the pool, were also selected for further study. eG1 was selected for further study since it expresses high levels of markers associated with an epithelial state, including keratins, the epithelial basement membrane protein *Lamc2*, and *Lgr4*, a regulator of mammary gland development and stem cell activity^29^.

**Fig. 3:**
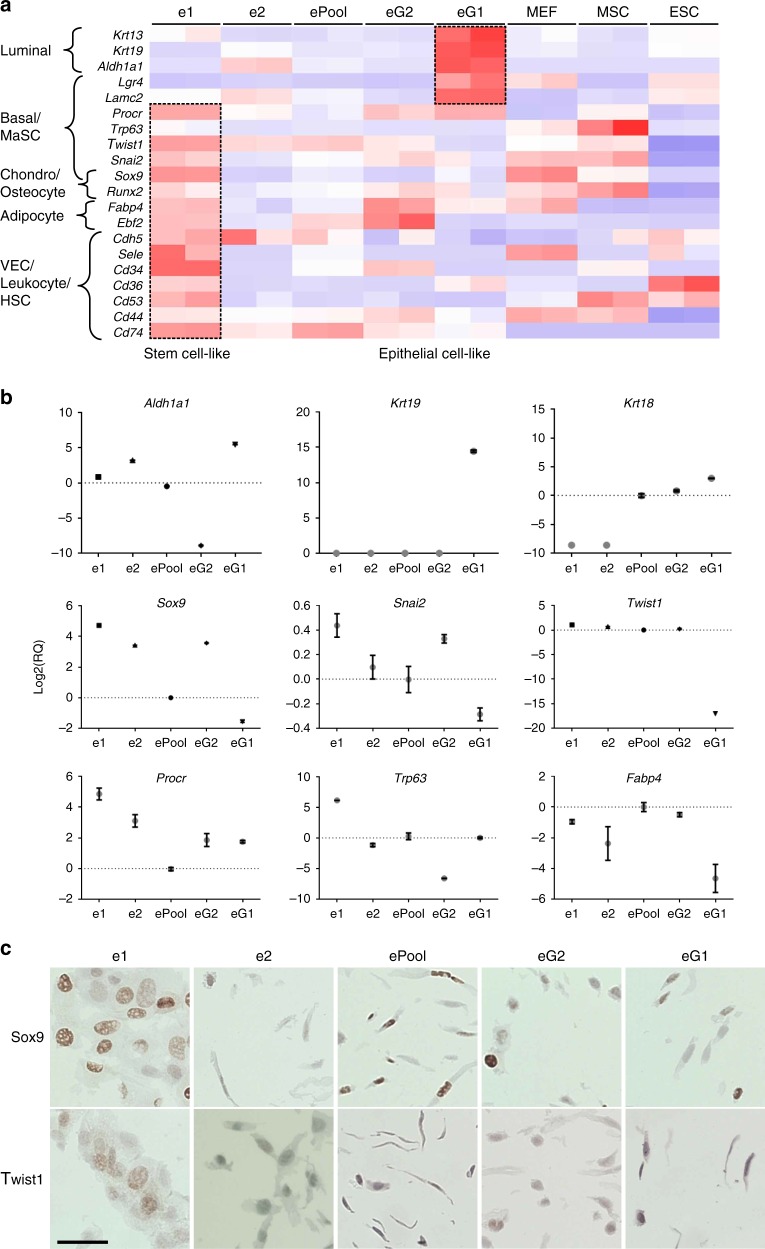
Progenitor and lineage-associated marker expression in select eMPCs. **a** Heatmap depicting stem cell and epithelial marker expression in four eMPC selected for further characterisation. **b** qRT-PCR for *Aldh1a1*, *Krt19*, *Krt18*, *Sox9*, *Snai2*, *Twist1*, *Procr*, *Trp63*, *Fabp4* in eMPC clones. Relative quantification (RQ) of each sample is normalised to that of ePool and shown in log 2 plot with error bar of triplicates (*n* = 3, mean ± s.e.m.). **c** Immunohistochemistry staining of Sox9 and Twist expression in eMPC. Scale bar, 200 μm. eMPC, embryonic mammary progenitor cell; MEF, mouse embryonic fibroblast; MSC, mesenchymal stem cell; ESC, embryonic stem cell; e1 and e2, single-cell-derived clones from GFP− cells; eG1 and eG2, single-cell-derived clones from GFP+ cells; ePool, pool of eMPCs; MaSC, mammary stem cell; VEC, vascular endothelial cell; HSC, hematopoietic stem cell

In total, two clones expanded from GFP+ cells (eG1 and eG2) and the two clones expanded from GFP− cells (e1 and e2) lines, along with the ePool, were used for further characterisation based on their distinct morphologies, transcriptome profiles and cluster analysis results (Fig. 3). The two GFP− cell lines (e1, e2) do not cluster together. However, single GFP+ and GFP− cells were expanded prior to other characterisation including RNA-sequencing, and these cell populations contained a mixture of both GFP+ and GFP− cells (Supplementary Fig. 1C).

We selected a subset of markers that have direct links to stem cell biology and confirmed the RNA-sequencing profiles using real-time quantitative reverse transcription-PCR (qRT-PCR) (Fig. 3b). We also used immunohistochemistry (IHC) to confirm expression of Sox9 and Twist1 protein in specific eMPC clones (Fig. 3c). Sox9 and Twist1 protein expressions were discordant with the RNA expression results, but mismatch between transcriptional and translational levels is a frequently occurring phenomena^30^.

### eMPCs harbour varying capacities for differentiation into the mesodermal lineage

**Fig. 4:**
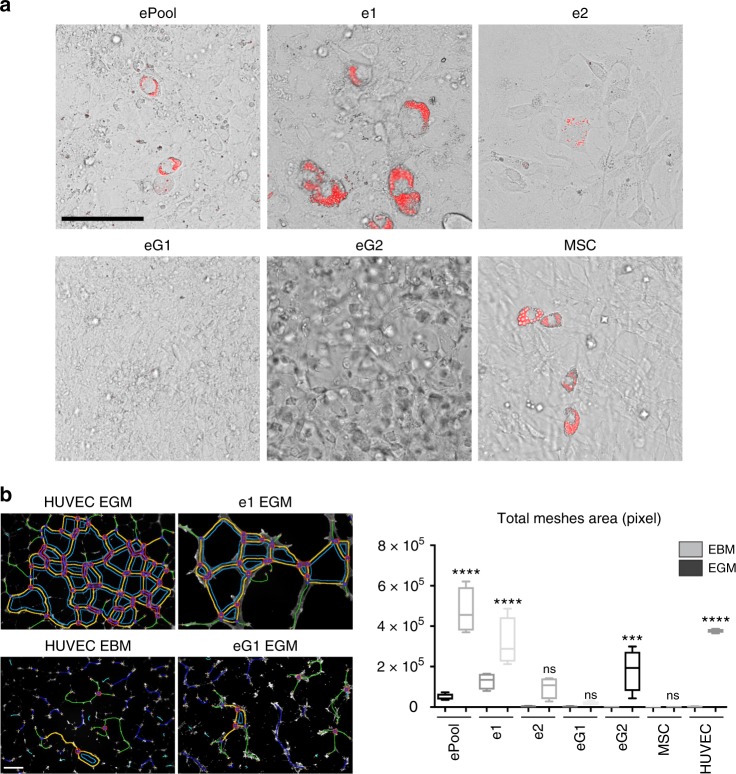
eMPCs harbour varying potential for differentiation into mesodermal lineage. **a** In vitro differentiation of eMPC clones and positive control (MSC) with adipogenic stimuli. Neutral lipid staining merged with bright-field image shows accumulation of lipid droplets (red) within cells after 6 days of induction. Scale bar, 100 μm. **b** Vasculogenesis assay results of eMPCs and positive control (HUVEC). Tubes formed in medium with growth factors (EGM) and without growth factors were stained with Calcein AM (white) after 24 h incubation. The total area of networks (blue, yellow and green lines) was analysed and presented in box plots with whiskers denoting minimum and maximum values (*n* = 4 mean ± s.e.m.). Statistical significance was computed using one-way analysis of variance (ANOVA) and Dunnett’s multiple comparisons test as ****P ≤ 0.0001, ***P ≤ 0.001, NS = no significance. Two-tailed *P* values are ePool EGM 0.0001, e1 EGM 0.0001, e2 EGM 0.1607, eG1 EGM 0.9994, eG2 EGM 0.0005, MSC EGM 0.9999 and HUVEC EGM 0.0001, when compared to control HUVEC EBM. Scale bar, 200 μm. eMPC, embryonic mammary progenitor cell; MSC, mesenchymal stem cell; HUVEC, human umbilical vein endothelial cells; EGM, endothelial cell growth media; EBM, endothelial basal growth media

We examined the capacity of eG1, eG2, e1, e2, and the ePool to differentiate into mesodermal lineages. Cells were exposed to differentiation media, previously reported to induce MSCs toward adipocyte or endothelial cell lineages. Under adipogenic conditions, only e1 cells produced lipid-filled adipocytes in a substantial fraction of cells, indicating notable adipogenic ability, whereas eG1 and eG2 do not respond to adipogenic stimuli. (Fig. 4a). Under endotheliogenic conditions, e1, eG2, and the ePool formed tubular network comparable to control Human umbilical vein endothelial cells (HUVECs) cultured in endothelium growth medium (Fig. 4b). Both e1 and eG2 express a vascular progenitor cell marker *Cd34* prior to induction (Fig. 3a). Overall, these functional studies suggest that clone e1 is the most responsive of the four cell lines to mesodermal lineage differentiation cues.

### eMPCs are clonogenic and form distinct sphere morphologies

**Fig. 5:**
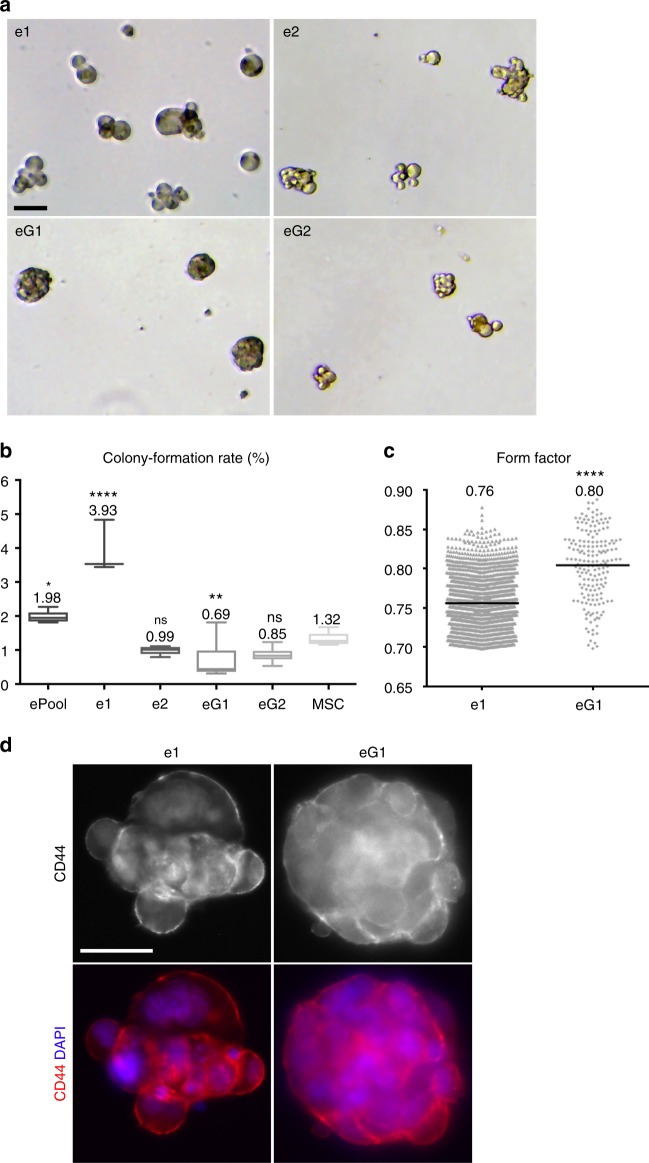
eMPC lines are clonogenic and display distinct acinar morphologies when grown as mammopheres in 3D culture. **a** Bright-field images of single-cell-derived spheres. Scale, 40 μm. **b** Colony formation ability presented in box plots with whiskers denoting minimum and maximum values showing number of cells (out of 10,000 cells plated) that gave rise to spheres in anchorage-independent cultures. Statistical significance was computed for each cell line compared to control (MSC) using one-way analysis of variance (ANOVA) and Dunnett’s multiple comparisons test, where ****P ≤ 0.0001, ** *P* ≤ 0.01, *P ≤ 0.05, NS = not significant. Two-tailed *P* values are ePool 0.0152, e1 0.0001, e2 0.3582, eG1 0.0092 and eG2 0.1107 when compared to control MSC (*n* = 3 mean ± s.e.m.). **c** Compactness of individual spheres presented where a circle with form factor = 1 is most condensed. eG1 forms more compact spheres compared to those of e1, which is also evident in images in **a**. Statistical significance was computed using unpaired *t* test, where two-tailed ****P = 0.0001. Bar indicates mean value ± s.e.m. *n* = 3. **d** Images of e1 and eG1 spheres stained by immunofluorescence with CD44 (red), an adhesion receptor expressed by basal mammary cells which mediates epithelial–stromal and cell–cell interactions and DAPI (blue). Scale bar, 50 μm. eMPC, embryonic mammary progenitor cell; MSC, mesenchymal stem cell

eG1, eG2, e1, e2 and the ePool were evaluated for mammary sphere-forming ability using the standard sphere formation technique. Each clone was cultured under sphere-forming conditions and formed spheres with highly distinct morphologies (Fig. 5a). All eMPCs formed spheres, although e1 had the highest sphere-forming rate (3.93%) and eG1 the lowest (0.69%) (Fig. 5b). Two clones (e1, eG1) were selected for more detailed functional assessments based on their distinct responses in functional assays and marker profiles. eG1 spheres show higher form factor, a measure of sphericity, when compared to those of e1, suggesting that eG1 spheres are relatively compact compared to the spheres formed from the other clones (Fig. 5c, d). e1 cells had limited engraftment ability when injected into cleared mammary fat pads (Supplementary Figure 3). No teratoma formation was observed, as would be expected to occur after xenografting of ESC cells.

### eMPCs form differentiated mammary acini in vitro

**Fig. 6:**
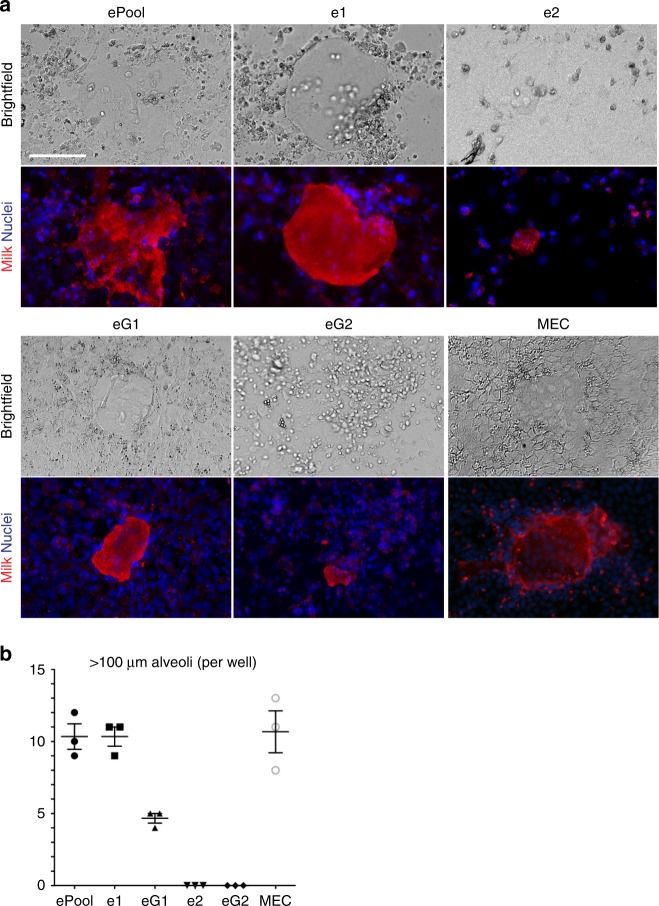
eMPCs have lactogenic potential. **a** Alveologenesis assay results for eMPC clones and positive control (MEC). Formation of alveolar-like structures and milk production was assessed by morphological changes using bright-field microscopy, as well as IF using anti-milk antibody (red). Scale bar, 40 μm. **b** Quantification of number of alveolar-like structures >100 μm. The plot is shown as count per 24-well (*n* = 3, mean ± s.e.m.). eMPC, embryonic mammary progenitor cell; MEC, mammary epithelial cell; ePool, pool of eMPCs; e1 and e2, single-cell-derived clones from GFP− cells; eG1 and eG2, single-cell-derived clones from GFP+ cells

eMPCs were evaluated for their ability to undergo functional alveolar differentiation by assessing cellular production of milk proteins by immunofluorescence staining with an antibody that detects milk-specific proteins including, β-casein, free secretory component, and lactoferrin. eMPCs were grown using conditions that induce lactogenic differentiation of mammary cells with prolactin. Under these conditions, e1, eG1, eG2 and ePool formed domes that produce milk protein, indicative of lactogenic differentiation (Fig. 6a). In these assays, e1 and ePool produced the greatest number of alveoli-like structures, with an alveologenic capacity to produce milk similar to postnatal MECs. eG1 and eG2 also showed lactogenic ability, but it was not possible to directly compare with the others since it was not possible to maintain the confluent cell culture needed for assessment at identical timepoints with this assay (Fig. 6b).

### *Sox9* ablation in e1 leads to reduced expression of Zeb1

**Fig. 7:**
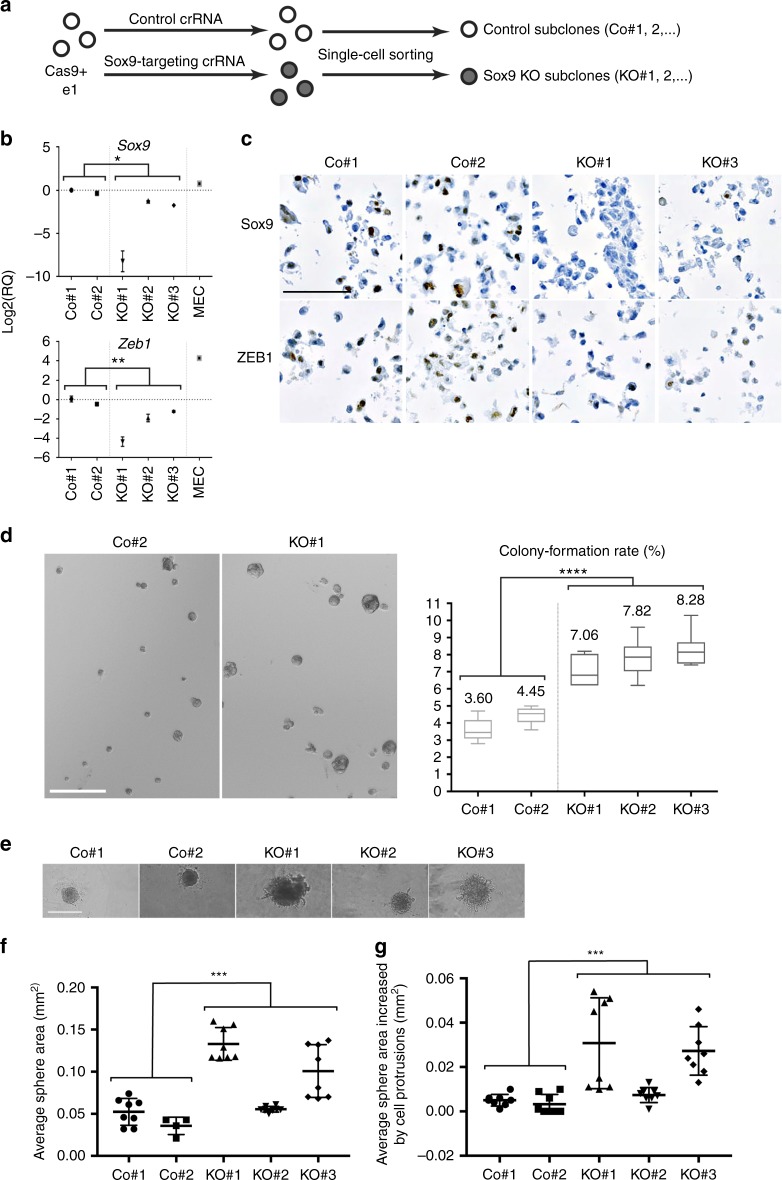
Loss of *Sox9* in e1 enhances colony formation ability and alters sphere morphology. **a** Schematic illustration for establishment of *Sox9*-targeted subclones from Cas9-expressing e1. **b** qRT-PCR for *Sox9* and EMT regulator, *Zeb1*. Each RQ is normalised to Co#1. Statistical significance between control group and *Sox9-*knockout group was computed using unpaired *t* test. **P ≤ 0.01, *P ≤ 0.05. Two-tailed *P* values are 0.0306 for *Sox9* and 0.0036 for *Zeb1*, respectively (*n* = 3, mean with 95% confidence interval). **c** Immunohistochemistry for Sox9 and Zeb1 showing Zeb1 expression is decreased by *Sox9* reduction. Scale bar, 200 μm. **d** Representative images of spheres grown in methylcellulose (left) and colony formation ability presented in box plots (right) with minimum and maximum values showing number of control or *Sox9-*targeted cells (out of 10,000 cells plated) that gave rise to spheres in anchorage-independent cultures. Statistical significance was calculated using unpaired *t* test. Two-tailed ****P = 0.0001 (*n* = 3, mean ± s.e.m.). Scale bar, 100 μm. **e** Representative images of spheroids grown in BME from control and *Sox9-*targeted cells. Scale bar, 400 μm. **f** Quantification of area of spheroids and **g** increase in area of protrusions from spheroids grown in BME from e1/control and e1*-Sox9-*KO cells (*n* = 8, mean + S.D.) Statistical significance was calculated using one-way analysis of variance (ANOVA) and multiple comparisons, ***P = 0.0002 in **f** and **g**. e1, embryonic mammary progenitor cell 1; Co, control, non*-*targeted cells; KO, knockout; *Sox9-*targeted cells; MEC, mammary epithelial cell

Mammary tissues originate from multipotent embryonic progenitors, which give rise to unipotent basal and luminal stem cells found and postnatal mammary tissues^31^. *Sox9* was selected for further study since it is amongst the earliest transcription factors expressed by cells within the embryonic mammary epithelium and its expression increases so that most cells express high levels of Sox9 by E14.5 (Supplementary Fig. 4)^22^. We assessed the role of *Sox9* in regulating embryonic mammary stem cell function using e1, a line that expresses a number of markers typically associated with stem/progenitor cells, including high levels of *Sox9*. CRISPR-Cas9 was used to create three independent subclones with gene deletions (or knockouts (KOs)) of *Sox9* derived from e1, e1/*Sox9*-KO#1, e1/*Sox9*-KO#2 and e1-*Sox9*-KO#3 (Fig. 7a).

We confirmed a reduction in *Sox9* levels in the three subclones with *Sox9* deletions compared to non-targeting guide control cells by qRT-PCR (Fig. 7b), IHC (Fig. 7c) and western blotting (Supplementary Fig. 5B). We also detected a marked reduction of expression of *Zeb1*, a key epithelial-to-mesenchymal transition (EMT) regulator, and concomitant with the level of reduction of Sox9 (Fig. 7b). Using IHC, we confirmed total loss of Sox9 expression in e1*/Sox9*-KO#1 subclone, but detected Sox9+ cells in 1–4% of e1/*Sox9*-KO#2 and e1/*Sox9*-KO#3 cells (Fig. 7c). Reduced detection of Sox9 in the three *Sox9*-KO subclones is correlated with reduction of Zeb1-expressing cells (Fig. 7c).

### *Sox9* ablation increases e1 clonogenicity

In non-targeting guide control cells derived from e1, e1/Co#1 and e1/Co#2, sphere-forming efficiency is similar in both the parental e1 clone and non-targeted e1 subclones (Figs.  5b and 7d). In all three independently derived e1 subclones with *Sox9* deletions, sphere-forming efficiencies increased by approximately twofold, indicating that deletion of *Sox9* in e1 enhances clonogenic ability, a measure of stem cell activity (Fig. 7d). When plated as multicellular aggregates on low-attachment plates, no change in spheroid morphology is observed (Supplementary Fig. 6). However, when spheroids are embedded with BME or Matrigel, the spheroids from *Sox9-*KO cells are larger and exhibit a greater number of cellular protrusions when compared to controls indicative of altered morphogenetic and enhanced migratory capacity (Fig. 7e, f and Supplementary Figure 6).

### *Sox9* ablation decreases response to lactogenic stimuli

**Fig. 8:**
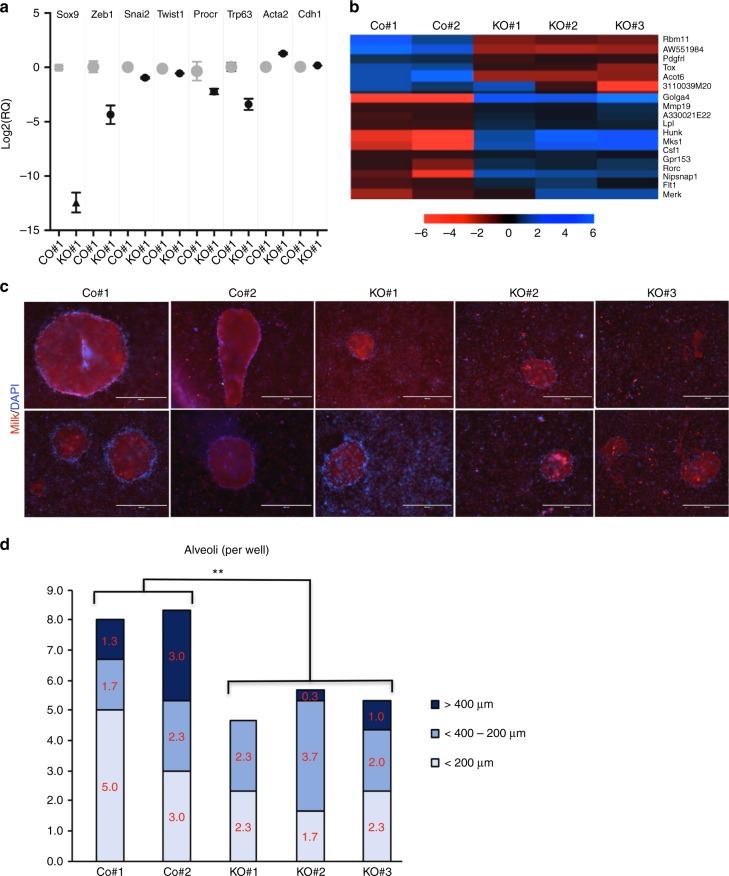
Effect of *Sox9* ablation on embryonic mammary progenitor cell fate and function. **a** qRT-PCR for *Sox9*, *Zeb1, Snai2*, *Twist*, *Procr*, *Trp63*, *Acta2*, and *Ecad* in e1/Co#1 and e1/*Sox9*-KO#1 cells. Relative quantification of each sample is normalised to that of e1/Co#1 and shown in log 2 plot with error bar of triplicates (*n* = 3, mean ± s.e.m.). **b** Heatmap from RNA-seq of e1/control and e1/*Sox9*-KO cells of genes that were significantly up- or down-regulated in all three e1/*Sox9*-KO subclones using both DESeq and Intensity Difference test. **c** In vitro alveologenesis assay results. Formation of alveoli-like structure and milk production was assessed by morphological changes using bright-field microscopy, as well as IF using anti-milk antibody (red) and DAPI. Scale bar, 200 μm. **d** In vitro alveologenesis assay results. Quantification of number of alveoli <200 μm, 200–400 μm, and >400 μm per well. Statistical significance between control group and *Sox9-*knockout group was computed using two-tailed paired *t* test. *P* = 0.0044. (*n* = 3). **P < 0.01 in **d**. e1, embryonic mammary progenitor cell; Co#1/#2, control #1/#2, non*-*targeted e1 cells; KO#1/#2/#3, knockout #1, #2, #3; *Sox9-*targeted e1 cells.

We assessed expression of several stem/lineage markers in e1/*Sox9*-KO#1, which lacks detectable Sox9 when cells are stained by IHC. *Acta2* (*α-SMA*), a marker normally expressed by basal stem cells and differentiated myoepithelial cells, was increased when Sox9 was ablated. Levels of *Procr*, a marker for a rare basal mammary stem cell population and *Trp63*, a basal cell marker, were both decreased, suggesting profound alterations of the basal features of these cells (Fig. 8a). We found other EMT regulators including *Snail2/Slug* and *Twist* were expressed at lower levels in cells lacking Sox9, whilst *E-cadherin* levels, an epithelial marker, were slightly increased, consistent with a reduced EMT signature that would be expected from reduced Zeb1 levels.

RNA-sequencing analysis confirmed that *Sox9* transcripts were not completely deleted from the three e1*/Sox9-*KO subclones. This was anticipated since the targeting strategy does not remove the 5′ transcript, including ATG start codon. e1/*Sox9-*KO#1 expresses the lowest *Sox9* levels compared to e1/*Sox9-*KO#2 and -KO#3, which show reduced *Sox9* levels compared to control cells (Supplementary Fig. 7). A small number of genes were consistently up- and down-regulated in all three e1/*Sox9-*KO lines when compared to e1/control subclones in transcriptomic analysis (Fig. 8b). One is *Csf1*, which promotes postnatal mammary stem cell activity, and is expressed at higher levels in all *Sox9-*deficient e1 subclones. Two clusters of interest were identified after clustering of all hits identified based on either DESeq or Intensity Difference tests. One group includes *Sox9*, whilst the other group displays the opposite expression pattern. e1/*Sox9*-KO#1 and e1/*Sox9*-KO#3 clustered more closely together than with e1/S*ox9*-KO#2, and both lines displayed pronounced changes in morphologies when cultured in 3D, so these two lines were analysed together to identify genes consistently modulated in these e1/*Sox9-*KO cells compared to the control cells. Genes associated with innate immunity, cholesterol biosynthesis, cell fate are down-regulated, while genes regulating cell adhesion, Rap1 signalling and kinase activity are upregulated in e1/*Sox9-*KO#1 and e1/*Sox9*-KO#3 cells, identifying potential regulators of the morphological changes observed when cultured in 3D (Supplementary Data 3).

Results from lineage tracing studies have shown that Sox9+ cells contribute to alveologenesis^32^. We therefore investigated the ability of e1/*Sox9*-KO cells to form fully differentiated mammary acini and secrete milk using an in vitro assay. Fewer alveoli-like structures derived from e1/*Sox9-*KO cells formed from all three e1/*Sox9-*KO lines and these were smaller and ill-defined structures compared to those formed from e1/control cells (Fig. 8c, d). Our results demonstrate a diminished ability of e1/*Sox9*-KO embryonic mammary cells to respond to lactogenic stimuli and undergo functional alveologenesis. These results indicate that Sox9 expression is required for embryonic mammary progenitor cells to attain a mature luminal progenitor cell phenotype capable of efficiently differentiating to form fully functional alveoli.

## Discussion

In this study, we have established 18 novel, cultivable, embryonic mammary progenitor cell lines (eMPCs). eMPCs will be a valuable resource for studying molecular regulators of normal breast development and understanding mammary cell fate and its plasticity. Based on gene expression and PCA of the eMPCs, we identified three major clusters. One cluster (marked in yellow in Fig. 2 and 11 out of the 16 clones) is likely to represent mammary epithelial progenitor cells in an undifferentiated, plastic state since these cells express high levels of several stem cell makers; another cluster of three clones (marked in green in Fig. 2) is likely to be representing more differentiated epithelial mammary progenitor cells since cells from this cluster express higher levels of keratins. The third cluster of two clones and the pool (marked in magenta in Fig. 2) appears to be composed of less plastic progenitor cells with restricted developmental potential. Four of the 18 cell lines were selected for use in plasticity assays to represent the three major clusters and included both GFP− clones we had derived from single GFP− cells, as well as 2 of the 16 GFP+ clones derived from single GFP+ cells. Although GFP+ expression was retained within the epithelial-appearing component after sustained organoid culture of the micro-dissected mammary organ, GFP expression is variable after expansion in 2D culture such that both GFP+ and GFP− cells are present in all eMPC lines, and we cannot assume that GFP+ clones are derived from embryonic mammary epithelial cells or that GFP− cells represent mesenchymal cells. We cannot exclude the possibility that GFP+ clones could have originated from other GFP+ cells present within the embryo, although our dissection protocol should exclude this possiblilty^21^. In addition, we observe a small percentage of cells that lack GFP expression within the mammary primordial epithelium.

Embryonic mammary lineage commitment is not completely understood^33^. It is still not clear how plastic or committed embryonic mammary cells are after removal from their native microenvironment. A subset of clones appeared to give rise to cell lines with mammary epithelial progenitor features and other clones appear less differentiated and more plastic. Another small group clustered together with the ePool, suggesting that it consisted of a mixture of cell types. Heterogeneity exists between the clone profiles and their properties. This may reflect different levels of commitment of individual cells to the mammary lineage or loss of mammary epithelial phenotype. In addition, stem cells exist in a variety or spectrum of cell states that can interconvert^34,35^, so the observed heterogeneity could represent lines comprised of various components of a mammary progenitor spectrum.

During embryonic development, tissues reshape during a combination of morphogenetic processes. Tissue compaction is a morphogenetic process by which a tissue adopts a tighter structure. Both cell adhesion and cell contraction play roles in tissue compaction^36^. Of the four clones assayed, only eG1, a line that expresses keratins, as well as other adhesion regulators (including protocadherins, integrins, ECM ligands) undergoes compaction. eMPCs are clonogenic and form mammary acini in vitro. All four of the eMPC lines tested can be induced to secrete milk with the appropriate hormonal stimulation (although e2 and eG2 with low efficiency), which demonstrates these cells have been committed to the mammary lineage, an event that occurs during embryonic development^6^. Clone e1 displayed limited ability to engraft in cleared mammary fat pads; this is consistent with other reports that found dissociated mid-gestation stage mouse mammary cells have very low stem cell activity when assessed using the cleared fat pad assay^12^. Baseline sequencing permits monitoring for changes after passage and we have been able to recluster eMPC cells together after early and later passages when re-sequenced, suggesting that these cells can be maintained without substantial changes.

Sox9 is amongst the earliest transcription factors expressed by cells within the prenatal mammary epithelium^22^. Sox9 is also expressed by basal and luminal oestrogen receptor (ER)—postnatal mammary epithelial cells^32,37^. Sox9 is thought to act together with Slug to confer a stem cell state to postnatal mammary epithelial cells^18^. Regulation of prenatal and postnatal mammary stem cell function by *Sox9* produces distinct effects on clonogenic ability. In contrast to our finding of increased sphere-forming ability in e1 after *Sox9* deletion, knockdown of *Sox9* in primary postnatal mouse MECs causes a marked reduction in organoid-forming ability, reduced *Slug* levels, and diminishes gland-reconstituting activity^18^. Impaired ability to undergo EMT is likely to be a common feature in all *Sox9*-deficient mammary cells. Mammary stem cell function is highly impacted by *Sox9*, but cell context also appears to be an important factor, including developmental stage and state. A recent study shows a role for SOX9 in the regulation of hormone resistance in breast cancer^38^, highlighting the relevance of embryonic mammary factor expression in breast cancers and the need for further investigations into their ability to modulate cell states.

Cell state is thought to influence epithelial cell phenotype and plasticity^39^. Mature postnatal MECs express keratins, indicative of being in an epithelial state (E). Loss of Sox9 and ability to undergo EMT in postnatal MECs should push them towards a more highly epithelial state, which, in theory, would have less stem cell activity according to several recent models^40^. Stem cell activity is thought to be highest when cells are in an intermediate epithelial/mesenchymal state^35^. Deleting *Sox9* from e1 leads to reduced Zeb1 levels and reduced propensity to undergo EMT. As a result, e1/ *Sox9-*KO cells would be expected to shift from the embryonic mesenchymal (M) state towards epithelial to an intermediate hybrid M/E state, which, in theory, should have more stem cell activity and mixed epithelial (adhesive) and mesenchymal (migratory) properties that confer ability to move collectively as clusters (Supplementary Fig. 8)^41,42^. Our results clearly show that there are profound differences between the effects from loss of *Sox9* function on clonogenic ability in embryonic mammary progenitor cells compared to postnatal MECs, which exist in relatively high epithelial cell states. Despite these differences, these findings are consistent with current models of EMT/MET mediation of cell state in regulating stem cell function.

Analysis from lineage tracing data indicated that Sox9 marks luminal progenitors that give rise to alveolar progenitors^32^. Fewer alveoli-like structures formed from e1/*Sox9*-KO cells and were smaller than control alveoli, which is consistent with a requirement for active Sox9 signalling in luminal progenitors to produce functional alveoli. *Sox9* is required for luminal progenitor cell survival, but basal/myoepithelial cells can withstand loss of *Sox9* in studies using a conditional deletion of *Sox9* from the mammary gland using mouse mammary tumour virus (MMTV)-Cre^37^. Mice with *Sox9*-deficient mammary glands could lactate normally, but mosaic MMTV-Cre mediated gene deletion was reported, so it is likely that sufficient numbers of Sox9+ luminal progenitors were retained to produce functional alveoli that had undergone terminal differentiation^37^. Our results support a model in which Sox9 is required for the specification of mammary progenitor cells from early embryonic stages, with postnatal development as a key regulator of mammary gland development and luminal progenitor homeostasis and maintenance.

Our study reveals complex interactions that underlie mammary lineage regulation, cell state and progenitor cell activity. Plasticity, defined as the ability of cells to dynamically change cell state, is crucial for many processes during mammary gland development and tissue homeostasis. Reversible EMT is central to tissue development, epithelial stemness and cancer metastasis. SOX9 has been linked to all three of these processes^18,37,43^.

Cell context is key in studies of mammary cell function and embryonic cells are highly plastic and multipotent, giving rise to both basal and luminal postnatal mammary cells. Cancer progression involves the loss of differentiated phenotype and acquisition of progenitor/stem cell features, which have recently been put forward as a hallmark of cancer^44^. These cell lines will be an invaluable resource to explore unresolved questions related to biology of mammary progenitor and stem cells, including those relevant to the origin of breast cancer.

## Methods

### Animal experiments

All animal work was carried out under UK Home Office project and personal licences following local ethical approval from The Institute of Cancer Research Ethics Committee and in accordance with local and national guidelines. Female mice (*Mus musculus*) were used for all experiments except breeding. Immortomouse and severe combined immune deficiency (SCID)/Beige mice were purchased from Charles River (Harlow, UK). *s*-*SHIP-GFP* mice were a gift from the late Professor Larry Rohrschneider (Fred Hutchinson Cancer Research Center, Seattle, WA, USA). Mice were housed in individually ventilated cages (IVCs) on a 12 h light/dark cycle and received food and water ad libitum. Mouse genotyping for detecting *Immorto* and *s-SHIP-GFP* transgenes was performed at Transnetyx (Cordova, TN, USA) and the results were used for colony maintenance.

### Isolation and culture of eMPC

Immortomouse were backcrossed with C57BL6/J (B6) for four generations. The immortomouse and *s-SHIP-GFP* mice were bred to obtain *Immorto:s-SHIP-GFP* embryo. E12.0-stage *Immorto:s-SHIP-GFP* embryo was dissected to isolate only the organ of mammary primordium number three as a microtissue, using Dumont #5 forceps and Tübingen Spring Scissors (Fine Science Tools GmbH, Heidelberg, Germany) using a Leica M205FA fluorescent stereomicroscope (Leica, Wetzlar, Germany). The remaining embryonic tissue was used for genotyping the *Immorto* transgene. Microtissue was immediately embedded into 200 μl of ice-cold Cultrex BME type 1, stem cell qualified, growth factor reduced (3434-005-02, AMS Biotechnology Ltd, Abingdon, Oxford, UK) within a well of a 48-well plate. The matrix-embedded tissue was transferred into CO_2_ incubator equilibrated to 33 °C, 5% CO_2_ atmosphere for 3 h. Culture medium was overlaid on the solidified BME during the pre-incubation. Culture medium contains Mesencult MSC basal medium for mouse (05501), Mesencult Stimulatory Supplements for Mouse (05502), 2mM l-glutamine (17100; all from STEMCELL Technologies UK Ltd, Cambridge, UK), 5U/ml mouse IFNγ (315-05, PeproTech EC Ltd, London, UK), and 0.1 mg/ml primocin (ant-pm1, Source Bioscience, Nottigham, UK). eMPCs migrated and proliferated within BME from the *Immorto:s-SHIP-GFP* microtissue for 1 month. Cells were harvested by trypsinisation and further expanded on 2D dishes precoated thinly with BME, and referred to henceforth as eMPC pool, abbreviated as ePool.

### Generation of eMPC clones

Fluorescence-activated cell sorting: ePool cells were harvested from the culture dish and adjusted to the concentration of 2 × 10^6^ cells/ml with phosphate-buffered saline (PBS). To stain apoptotic and necrotic cells, cells were stained with DAPI (4′,6-diamidino-2-phenylindole) at a final concentration of 1μg/ml for 5 min at room temperature prior to cell sorting. ePool cells were passed through a 40μm filter and sorted using BD Aria Fluorescence-Activated Cell Sorter (BD Biosciences, Oxford, UK). Live single ePool cells were positively gated using their forward and side scatter profiles (Supplementary Fig. 1B). ePool population with high s-SHIP-GFP expression and ePool GFP− population were subsequently gated based on green channel profile.

Clone derivation from single cells: Live single ePool GFP+ and ePool GFP− cells were individually sorted into 96-multiwell plates. The multiwell plates were precoated with either Cultrex BME or Matrigel, growth factor reduced (734-0268, VWR International, Lutterworth, Leicestershire, UK) and filled with eMPC culture medium. The single cells on multiwell plates were incubated at CO_2_ incubator equilibrated to 33 °C, 5% CO_2_ atmosphere without changing medium for a month. eMPC clones proliferated for a month, after which they were trypsinised and transferred to dishes precoated with BME for further expansion. Only two GFP− eMPC clones were grown from a Matrigel-precoated multiwell plate and named as e1 and e2 (Fig. 1b). Fourteen GFP+ eMPC clones were recovered from two Matrigel-precoated multiwell plates, which are named as eG1, eG2, eG2E9 and so on, depending on well position. Only one GFP+ eMPC clone, eG1B3, was recovered from a BME-precoated plate, suggesting that Matrigel is preferable for initial clonal cell expansion.

### Cell culture

The following mouse C57BL/6 (B6) cell lines were purchased and cultured for RNA isolation according to the manufacturer’s instructions: BMMCs, (c57–6271F, Caltag Medsystems, Buckingham, UK); ESCs (SCRC-1002, LGC Standards, Middlesex, UK); MEFs, untreated, (GSC-6002, AMS Biotechnology Ltd); mesenchymal stem cells, (MUBMX-01001 Cambridge Bioscience, Cambridge, UK). B6 postnatal mammary epithelial cells were isolated as described^45^. The mammary epithelial fraction was further purified using EasySep Mouse Epithelial Cell Enrichment Kit according to the manufacturer’s instructions (19758, STEMCELL Technologies UK). HUVECs were cultured according to the manufacturer’s instruction (191027, Lonza, Basel, Switzerland).

### Total RNA isolation and quality control

Embryonic mammary tissues (embryonic mammary primordial epithelium (MPE), embryonic mammary stroma (includes MM and FPP), and surface epithelium (Epi) were microdissected from E12.5 B6 mice following the protocol described in ref. ^46^. Total RNA from cells and mammary tissues were extracted using RNeasy Plus Micro Kit and RNeasy Mini Kit (74034 and 74104, respectively, Qiagen, Manchester, UK), RNA concentration and genomic DNA (gDNA) contamination were determined using Qubit 2.0 Fluorometer and accompanying kits (Thermo Fisher Scientific, Waltham, MA, USA). RNA samples highly contaminated with gDNA for more than 10% of RNA were treated with TURBO DNA-free Kit (AM1907, Thermo Fisher Scientific) and concentrated using RNA Clean and Concentrator-5 (R1015, Zymo Research, Irvine, CA, USA). All samples were assessed for RNA quality using Agilent 2100 Bioanalyzer system (Agilent Technologies, Cheshire, UK) and confirmed that RNA Integrity Number was >9.0.

### RNA-sequencing

Complementary DNA (cDNA) library preparation was carried out at Oxford Genomics Centre, The Wellcome Trust Centre for Human Genetics using PolyA+ RNA enrichment method for total RNA from cultured cells and SMARTer method for total RNA from embryonic mammary tissue, respectively. Messenger RNA fraction was selected from the total RNA before conversion to cDNA. Second-strand cDNA synthesis incorporated dUTP. The cDNA was end-repaired, A-tailed and adapter-ligated. Prior to amplification, samples underwent uridine digestion. The prepared libraries were size selected, multiplexed and quality checked before paired-end sequencing over three lanes of a flow cell. Amplified cDNA from embryonic mammary tissues were generated by the SMARTer Amplification Kit. The cDNA was end-repaired, A-tailed, adapter-ligated and amplified. The prepared libraries were size selected, multiplexed and quality checked before paired end sequencing on four lanes of a flow cell. Data were aligned to the reference genome, mm10, and quality checked.

RNA-sequencing files were submitted to ArrayExpress as accession [E-MTAB-6846, MTAB-6856, E-MTAB-6859]. FastQ files were truncated to a consistent length of 75 bp using trim galore v0.4.3 and were then aligned against the mouse GRCm38 genome assembly using hisat2 v2.0.5 using options --no-mixed and --no-discordant. Mapped positions with mapping quality score values of <20 were discarded. Gene expression was quantitated using the RNA-sequencing quantitation pipeline in the SeqMonk software v1.37.0 (https://www.bioinformatics.babraham.ac.uk/projects/seqmonk/) in opposing strand-specific library mode. For count-based statistics, raw read counts over exons in each gene were used. For visualisation and other statistics log 2RPM (reads per million reads of library) expression values were used. Differentially expressed genes were selected based on passing two statistical filters: the DESeq2 LRT with a cutoff of *p* < 0.05 following multiple testing correction and the SeqMonk Intensity Difference filter on log 2RPM values with a sample size of 1% of all genes and a cutoff of *p* < 0.05 after multiple testing correction. Hierarchical clustering was performed on per-gene median centred log 2RPM expression values using Pearson’s correlation. Gene cluster separation was performed by segmenting the tree at an *R* value of 0.5. Clusters containing <50 genes were discarded. PCA was performed on column-centred log 2RPM values without additional scaling.

The intensity difference test used a locally matched subset of 1% of genes based on average expression. From these a local standard deviation in log 2RPM difference values was calculated and used to calculate the probability of the cumulative distribution function for a normal distribution with this standard deviation, using the observed difference in the gene being tested. *P* values were corrected for multiple testing using Benjamini and Hochberg multiple testing correction.

### cDNA synthesis and qRT-PCR

cDNA library from total RNA was synthesised using QuantiTect Reverse Transcription Kit (205310, Qiagen). Comparative *C*_T_ (ΔΔ*C*_T_) real-time quantitative PCR was performed using Taqman Gene Expression assays as previously described^17^ on either ABI Prism 7900HT Sequence Detection System (Applied Biosystems, Foster City, CA, USA) or QuantStudio 6 Flex System (Thermo Fisher Scientific). *Actb* was used as an endogenous control and fold change normalised to a comparator sample was calculated. The assay probes are listed in Supplementary Data 4.

### Antibodies

Antibodies used for whole-mount immunofluorescence, IHC, immunofluorescence, and western blotting in this study are listed in Supplementary Data 4.

### Adipogenesis assay

eMPC and MSCs (positive control) were plated at 100% confluency in Osteogenic/Adipogenic Base Media (CCM007, Bio-Techne Ltd, Oxford, UK) containing 1 μg/ml insulin, 0.5 mM 3-isobutyl-1-methylxanthine and 0.25 mM dexamethasone (19278, I5879 and D2915 from Sigma, Dorset, UK, respectively). After 6 days, cells were fixed with 4% paraformaldehyde (PFA)/PBS and lipid droplets were detected by HCS LipidTOX™ Red Neutral Lipid Stain (H34476, Thermo Fisher Scientific) at 1:200 in PBS. Lipid-labelled cells were photographed using EVOS FL fluorescent microscope equipped with ×10 Plan LWD FL 0.30NA objective (Thermo Fisher Scientific).

### In vitro tube formation assay

Tube formation ability of eMPCs was examined following a published protocol^47^. Briefly, eMPCs, MSCs (negative control) and HUVECs (positive control) were plated at ~80% confluency using endothelial growth medium (EGM) (EBM-2 medium containing EGM-2 SingleQuot Kit, CC-3156 and CC-3162 from Lonza, respectively). The next day, 96-well plates filled with 50–80 μl Cultrex BME were prepared on ice without forming air bubbles and pre-incubated at CO_2_ incubator equilibrated to 37 °C, 5% CO_2_ atmosphere to allow matrix gelation. Cells were harvested by trypsinisation and re-suspended at the concentration of 1.5 × 10^5^ cells/ml in either EBM-2 or EGM-2 medium. One hundred microliters of single-cell suspension (containing total 15,000 cells) was gently added on the top of BME gel. After incubation for 24 h, cells were labelled with Calcein AM solution at the final concentration of 2 μM in EBM-2 medium and incubated in CO_2_ incubator for 30 min. Calcein AM-labelled cells were photographed using EVOS FL fluorescent microscope equipped with ×2 Plan LWD 0.06NA objective (Thermo Fisher Scientific). The fluorescent images were analysed to detect mesh structure using a developed package of ImageJ, Fiji^48^ (http://imagej.net/Fiji/Downloads), and its Plugin, Angiogenesis Analyser (Carpentier G., Angiogenesis Analyser for ImageJ (2012); https://imagej.nih.gov/ij/macros/toolsets/Angiogenesis%20Analyzer.txt). Cells were also tested for tube formation assay with EBM-2 medium containing 10 mM sulforaphane (angiogenic inhibitor, S4441, Merck KGaA, Darmstadt, Germany) as a control to observe its inhibitory effect on the assay.

### Mammary alveologenesis assay

Lactogenic ability of eMPCs was examined following an established protocol^49^. Briefly, eMPCs and MECs (positive control) were plated at 100% confluent in RPMI-1640 medium (21875, Thermo Fisher Scientific) containing 10% horse serum (16050-122, Thermo Fisher Scientific), 5 μg/ml insulin, 10 ng/ml epidermal growth factor (EGF) (236-EG-200, Bio-Techne Ltd), 10 mM HEPES ((4-(2-hydroxyethyl)-1-piperazineethanesulfonic acid) (H0887, Sigma), and primocin (Bioscience). On the following day, in order to withdraw the EGF and enhance differentiation, rather than cell proliferation, the cell medium was changed to EGF− medium containing 5% horse serum, 10 mM HEPES, and primocin in RPMI-1640 medium. After 3 days, mammary alveologenesis was induced by replacement with priming medium (EGF− medium containing 5 μg/ml mouse prolactin (757908, BioLegend, London, UK), 5 μg/ml insulin, and 0.1 mM dexamethasone). Cells were fixed with 4% PFA/PBS 6 days after induction and stained with anti-milk antibody and DAPI. Alveoli were photographed using EVOS FL microscope and the number of alveoli >100 μm per 24 well was counted manually.

For studies of e1 with reduced *Sox9* expression, the assay was modified since e1/*Sox9*-KO cells detached upon induction of lactogenic differentiation using the standard assay. Cells were plated around 80–90% confluence in MesenCult Expansion medium with MesenCult supplement, 200mM l-glutamine, 5U/ml IFNγ (PeproTech) and 0.1 mg/ml primocin. Once cells reached 100% confluence (on day 4), the cell medium was changed to RPMI-1640 medium containing 5% horse serum, 10 mM HEPES, and primocin. On day 5, mammary alveologenesis was induced with medium containing 5 μg/ml mouse prolactin, 5 μg/ml insulin, and 0.1 mM dexamethasone. Induction medium was replenished every 3 days. On day 11, cells were fixed with 4% PFA/PBS and stained with anti-milk antibody and DAPI. Alveoli were photographed using EVOS FL microscope and the number of alveoli in different size ranges were counted manually.

### Sphere-forming assays

A clonogenic mammosphere protocol and media were adapted for eMPCs^50^. Mouse EpiCult-B complete medium (05610, STEMCELL Technologies) was supplemented with 0.6% methylcellulose (HSC011, Bio-Techne Ltd), 20 ng/ml basic fibroblast growth factor (3139-FB-025/CF, Bio-Techne Ltd), 20 ng/ml EGF, 2% NeuroCult™ SM1 without Vitamin A, 10 μg/ml heparin, 1 μg/ml hydrocortisone (05731, 07980, and 07926, respectively, from STEMCELL Technologies UK Ltd), 10 μg/ml insulin, 5U/ml mouse IFNγ, and 0.1 mg/ml primocin. Trypsinised eMPCs and MSCs were passed through 40 μm filter to make absolute single-cell suspension and the cell number was adjusted to 2–4 × 10^5^ cells/ml. Ten thousand cells were added into 2 ml of the supplemented Epicult-B medium. Cells were pipetted thoroughly to be distributed homogenously within the viscous methylcellulose-containing medium and transferred into Corning ultra-low-attachment 6 well (10154431, Fisher Scientific, Loughborough, UK). Six-well plates were incubated within a humidified box at 33 °C incubator for eMPCs and 37 °C for MSCs, respectively. At day 7, sphere images were photographed and automatically scored using Single Colony Verification program in Celigo cytometer (Nexcelom Bioscience LLC, Lawrence, MA, USA).

### Spheroid growth in hydrogel

For analysing 3D morphology, e1/control and e1/*Sox9*-KO cells were seeded with 5000 cells/well onto Corning Costar Ultra-Low-Attachment 96-well plates (Corning Kennebunk ME, USA) filled with the eMPC culture medium. On day 3, 50 µl of Corning Matrigel Growth Factor Reduced Basement Membrane Matrix (356231) or Cultrex PathClear 3-D Culture Matrix RGF BME I (3434-005) was added into each well (except control wells). The medium was replenished every 3 days. On day 9, 50 µl of 1% agarose was added to the control wells. All spheroids were subsequently fixed with 4% PFA/PBS and were photographed using EVOS FL microscope.

### CRISPR/Cas9-mediated gene targeting of *Sox9*

We took advantage of lentiviral transduction of *Cas9* gene within eMPCs followed by transfection of pre-made CRISPR RNA (crRNA) and trans-activating crRNA (tracrRNA) for genetic ablation of *Sox9*, which were purchased from GE Healthcare (Little Chalfont, UK). Edit-R Lentiviral Blast-Cas9 Nuclease Particles (VCAS10128, Dharmacon, Lafayette, Colorado, USA) were transduced into e1 and Cas9-expressing e1 population was expanded with 10 μg/ml blasticidin (ant-bl-1, Source Bioscience, Nottingham, UK) from day 4 onward. A total of 2 × 10^5^ cells of blasticidin-resistant e1 were seeded on 6 well and on the next day treated with 5 μl DharmaFECT 1 Transfection Reagent (T-2001-02, Dharmacon) together with 5 μl of 10 μM Edit-R CRISPR-Cas9 Synthetic tracrRNA (U-002000-20, Dharmacon) and 5 μl of 10 μM *Sox9*-targeting crRNA or non-targeting crRNA. Cell populations with *Sox9* mutations were screened by Heteroduplex formation assay using QuickExtract DNA Extraction Solution (QE0905T, Qiagen, Peterborough, UK) and EnGen™ Mutation Detection Kit (E3321S, New England Biolabs, Herts, UK) according to the manufacturer’s instructions. We tested four different crRNA and the primer sets to detect mutation within each crRNA-binding region; details are provided in Supplementary Data 4C. Two cell populations (Sox9-01 and Sox9-02 in Supplementary Fig. 5A) that were verified for *Sox9* mutation were further sorted using BD Aria to develop single-cell-derived subclones. Three e1/*Sox9*-KO subclones, KO#1, KO#2, and KO#3, produced no Sox9 expression detectable by western blot (Supplementary Fig. 5B). These three e1/*Sox9*-KO subclones as well as two e1/control clones, Co#1 and Co#2, were used in this study.

### Mammary fat pad injection of e1

To allow bioluminescence imaging of e1 cells, cells were labelled with carrying red-shifted *Luciola Italica* luciferase transgene using lentivirus particles, RediFect Red-FLuc-Puromycin (CLS960002, Perkin Elmer, Buckinghamshire, UK). The transduced e1/Red-FLuc cells were selected with 5 μg/ml puromycin for 3 weeks. e1/Red-FLuc cells were harvested and re-suspended at a concentration of 1 × 10^6^ cells/ml in PBS. One hundred microliters of cells were injected into both the left and right inguinal mammary fat pad number 4- of 10-week-old SCID/Beige female mice. Mice were injected 100 μl of 15 mg/ml d-Luciferin (119222, Perkin Elmer) in PBS intraperitoneally and imaged after 5 min using IVIS Illumina II (Perkin Elmer). Engrafted mammary glands were harvested 1–2 weeks after mammary fat pad injections and fixed in 4% PFA in PBS for IHC.

### Statistical analysis

The data in the graphs are presented as mean and the standard error of the mean. The data were analysed by two-tailed analysis of variance, Student’s *t* test, or two-tailed, paired *t* test using the GraphPad Prism 7 software. *P* value  ≤ 0.0001 is considered as extremely significant (****), *P* ≤ 0.001 as highly significant (***), *P* ≤ 0.01 as very significant (**), *P* *≤* 0.05 as significant (*) and *P* > 0.05 as not significant (NS), respectively.

## Electronic supplementary material

Supplementary Information

Description of Additional Supplementary Files

Supplementary Data 1

Supplementary Data 2

Supplementary Data 3

Supplementary Data 4

Supplementary Data 5

## Data Availability

The authors declare that the data supporting the findings of this study are available within the article and its supplementary information files. All source data underlying the graphs and charts presented in the main figures is available in Supplementary Data 5.
