# Supplementary material for: Sox9 regulates cell state and activity of embryonic mouse mammary progenitor cells
Source: Commun Biol. 2018 Dec 13;1:228. doi: 10.1038/s42003-018-0215-3 (PMC6292906; doi:10.1038/s42003-018-0215-3)
Supplement: Supplementary file 1 — Supplementary Information [file 42003_2018_215_MOESM1_ESM.pdf]

Supporting Figures and Legends

Supplementary Figure 1

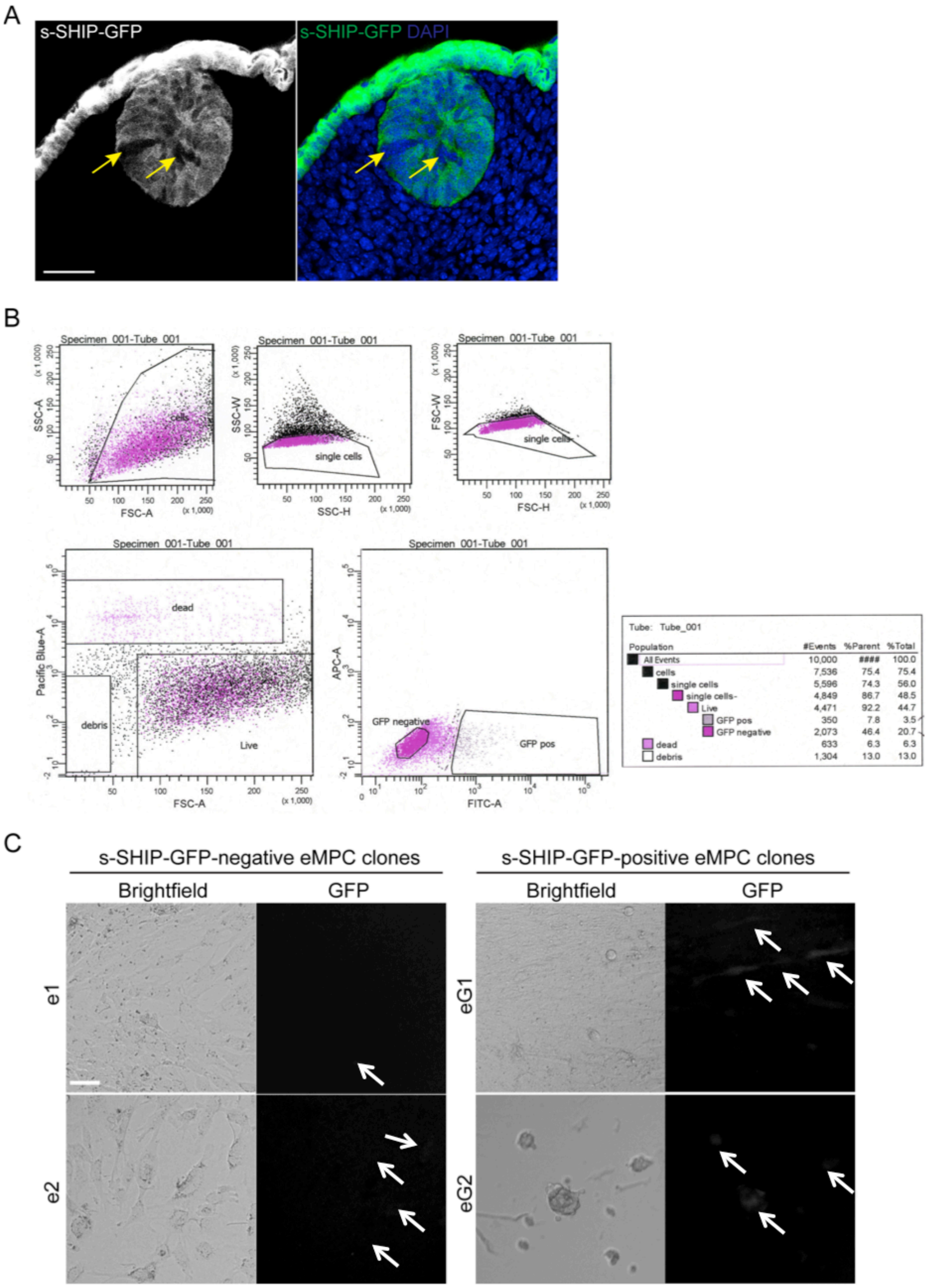

(A) Confocal image of E12.0 s-SHIP-GFP mammary organ stained with GFP and DAPI. Yellow arrows indicate GFP- cells within mammary primordium epithelium. Scale bar, 200 μm.

(B) Flow cytometry analysis of GFP expression in dissociated embryonic mammary progenitor cells isolated from microdissected *Immorto;s-SHIP-GFP* mammary organ from E12.0-stage embryo after culture on BME for one month.

(C) GFP expression in lines derived from either single GFP+ (eG1, eG2) and GFP- (e1, e2) embryonic mammary progenitor cells after expansion in 2D culture. White arrows indicate GFP+ cells. Brightfield images are included. Scale bar, 200 μm.

Supplementary Figure 2

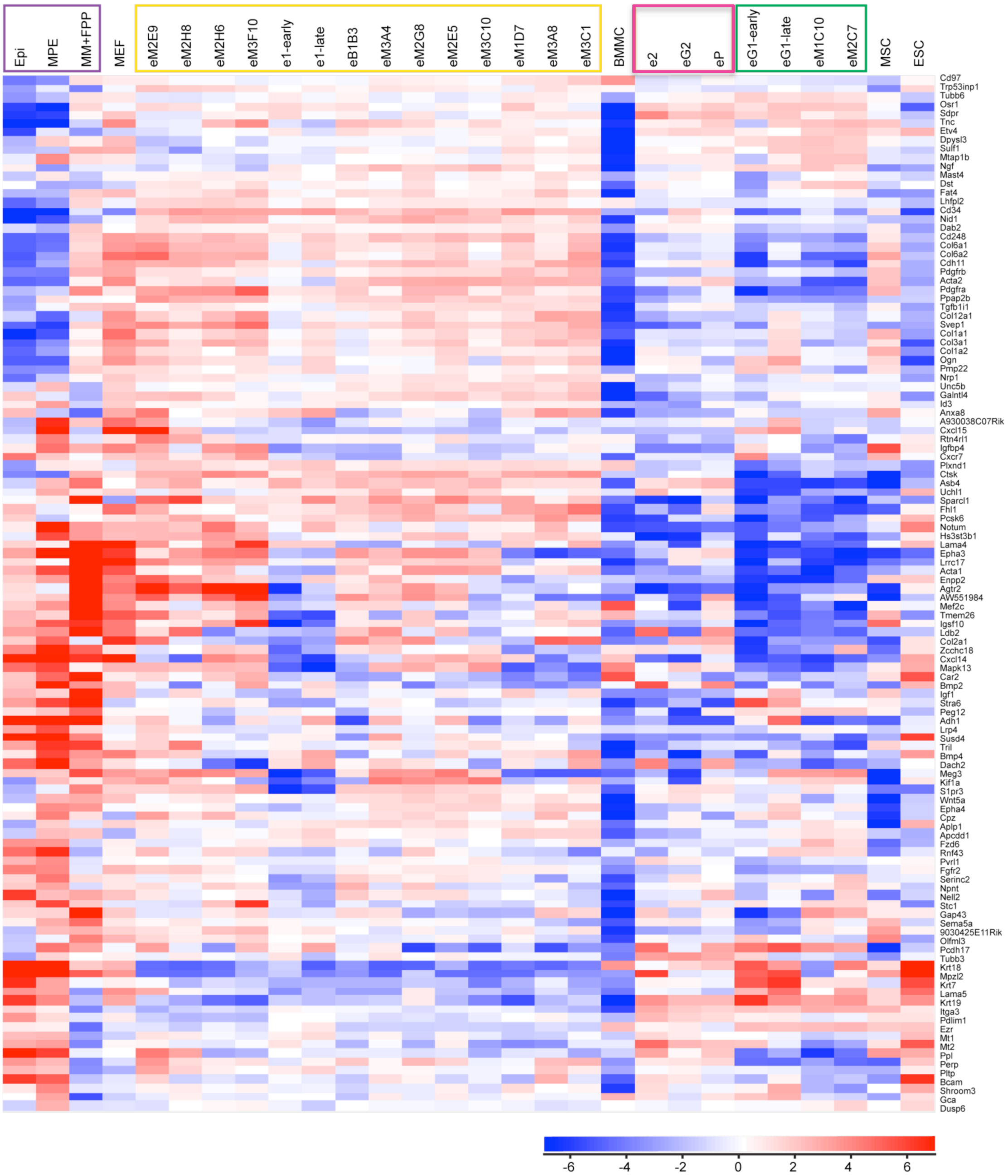

Heat map showing gene expression profiles of 118 genes used in principal component analysis (PCA) of eMPC cells, other stem cell types and embryonic mammary tissues. MPE, mammary primordial epithelium, Epi, epithelium, MM, mammary mesenchyme, FPP, future fat pad precursor. Early and late indicate clones that were sequenced at early (P=5) and later (P=13) passage.

Supplementary Figure 3

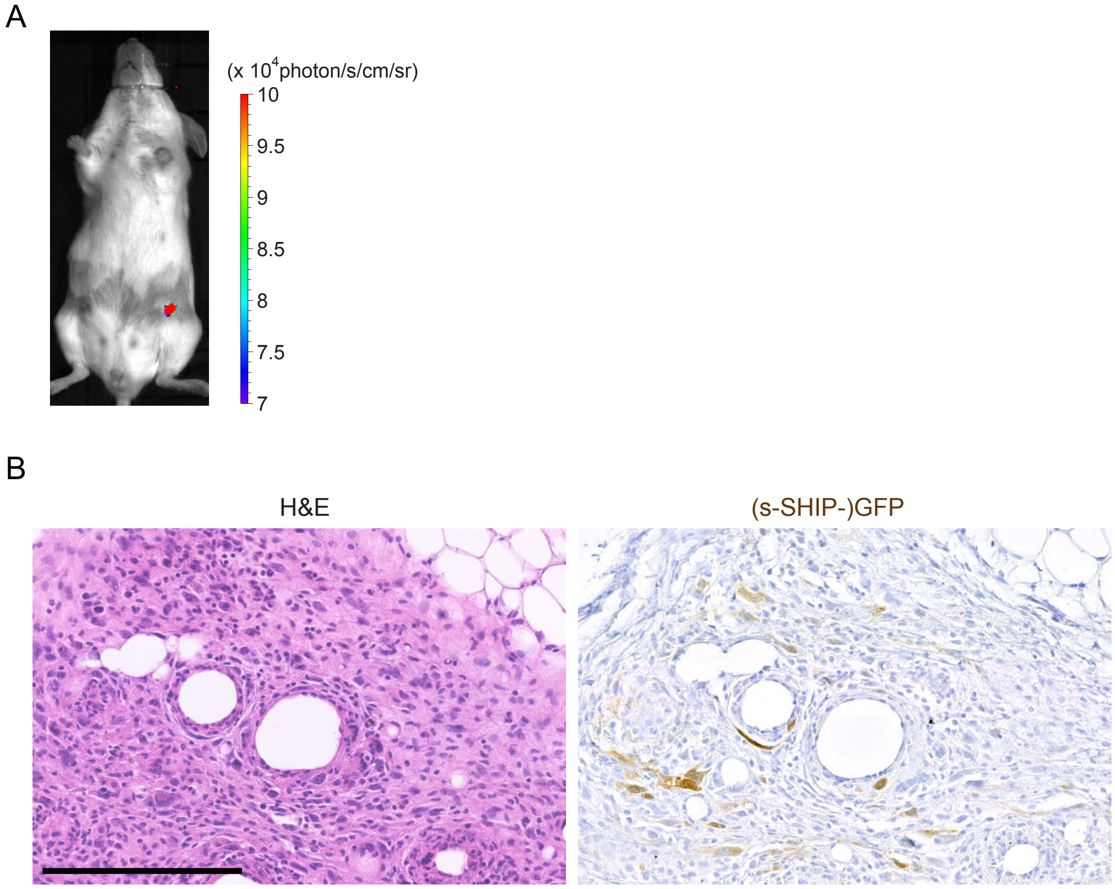

(A) IVIS results show limited engraftment of e1/Red-FLuc cells xenografted into SCID/Beige female mice. 2 out of 12 mammary gland xenografts displayed bioluminescence beyond one week of engraftment.

(B) Example of mammary gland from (A) that retained bioluminescence two weeks after grafting e1/Red-FLuc cells. Sections stained with H&E or GFP. Scale bar, 200 μm.

Supplementary Figure 4

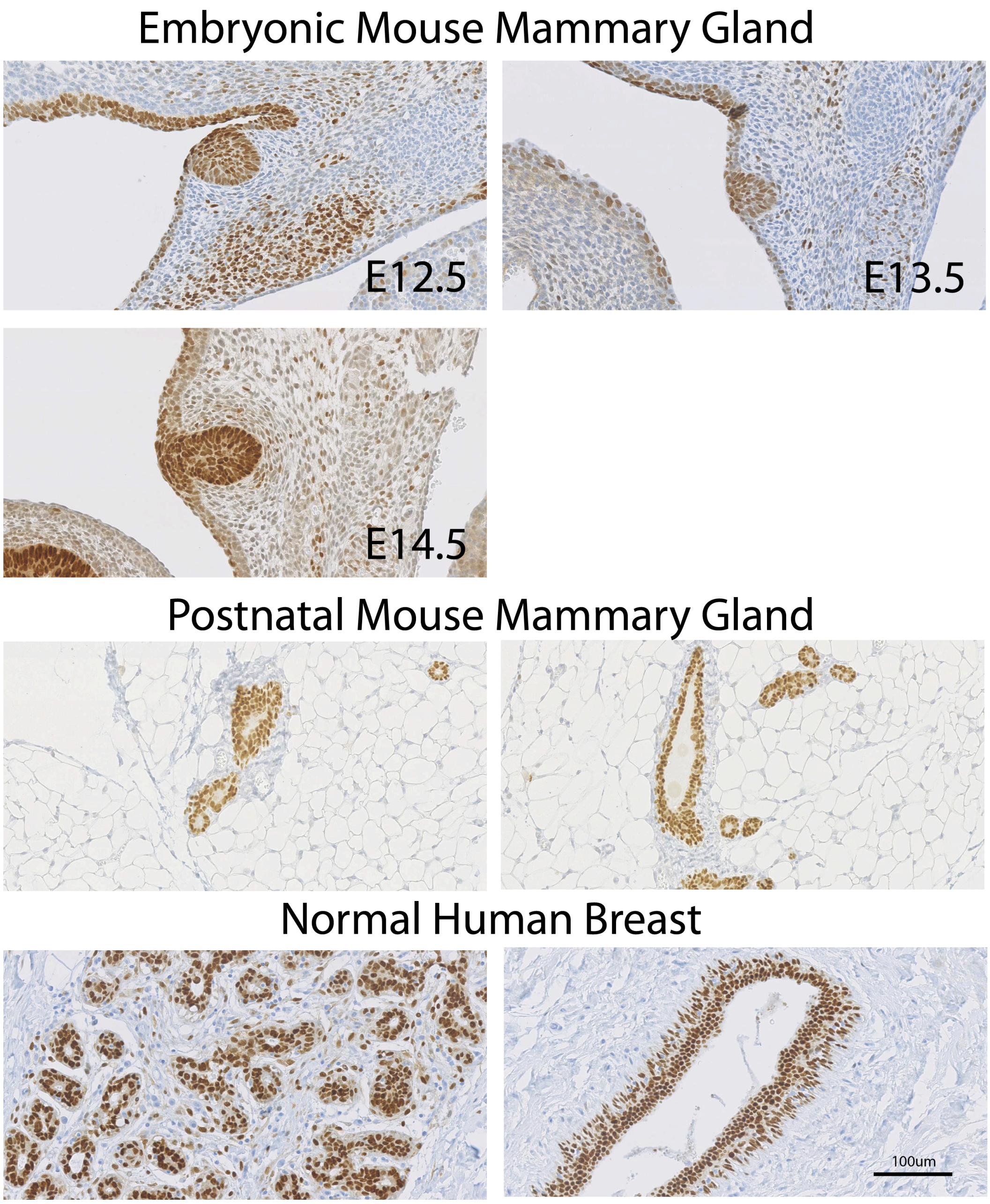

Sox9 staining of embryonic mammary organs from Balb/c mice at E12.5-, 13.5-, E14.5-stages, nulliparous postnatal (14-week) mammary gland 4, and human breast. Scale bar, 100 μm.

Supplementary Figure 5

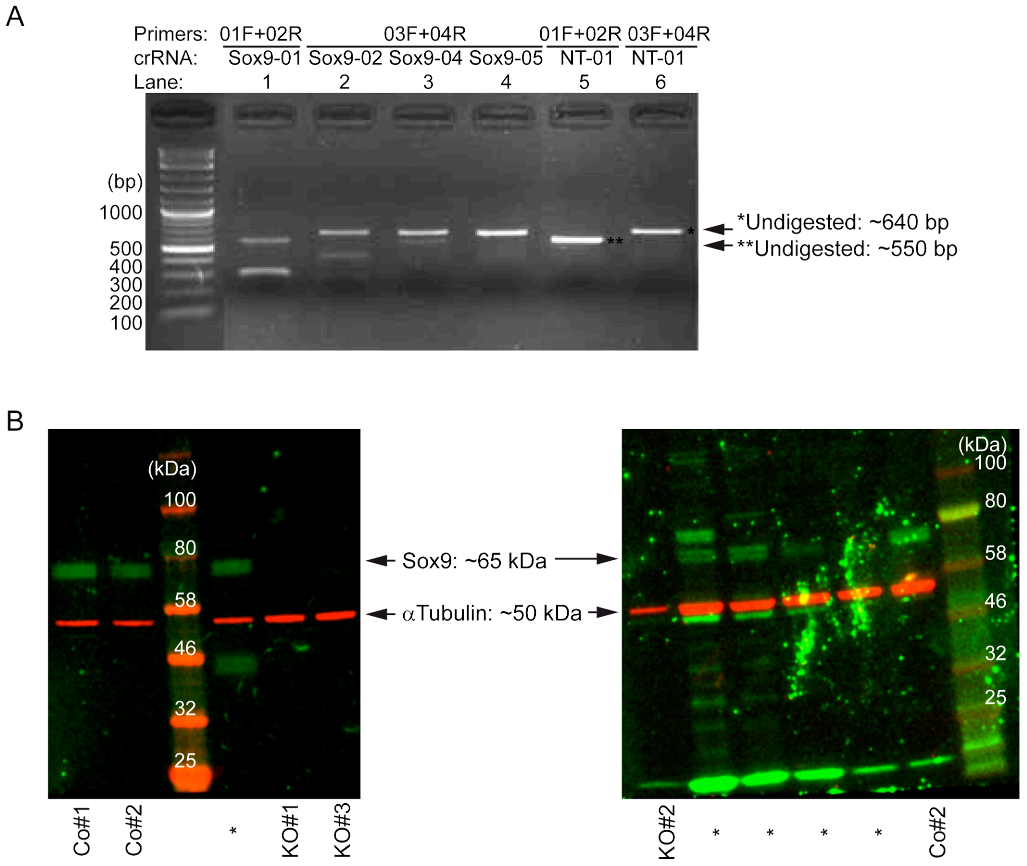

(A) Heteroduplex formation assay using the indicated primer sets to detect *Sox9* mutation in pooled cell populations (Sox9-01, Sox9-02, Sox9-04 and Sox9-05) after CRISPR-Cas9 mediated gene targeting. Digestion of PCR products from targeted cell populations was compared to the undigested PCR products obtained using non-targeted pooled cells (NT-01) and the indicated primer sets, revealing *Sox9* mutation in Sox9-01 and Sox9-02 pools.

(B) Near-infrared western blot analysis of Sox9 and Tubulin in two guide control (Co) and three knockout (KO) clones derived after CRISPR-Cas9 mediated gene deletion that were used in this study.

Supplementary Figure 6

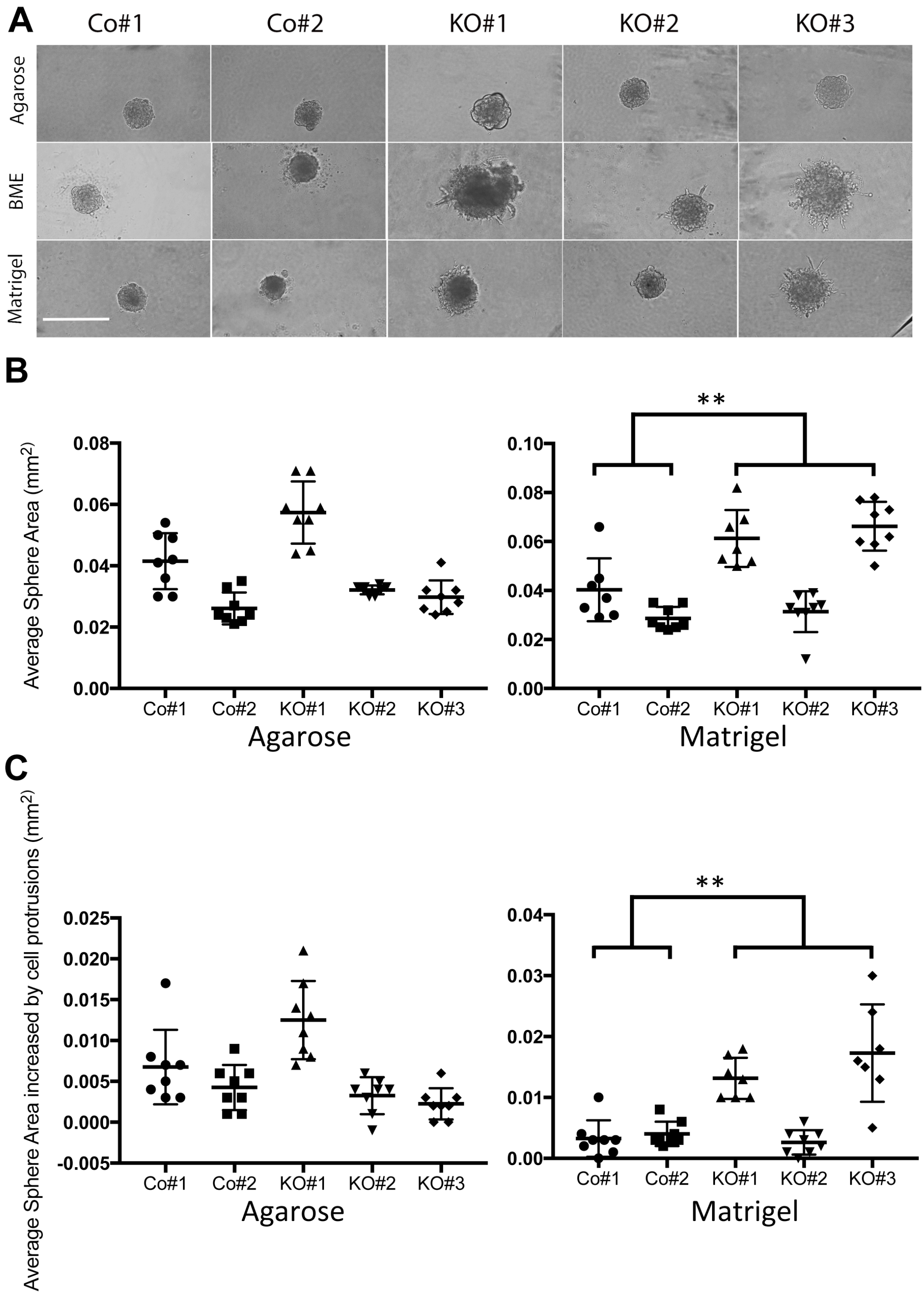

(A) Representative images of morphologies of e1/ control (Co) and e1/*Sox9*-KO spheroids grown in agarose, BME and Matrigel. scale bar, 400 μm.

(B) Quantification of area of spheroids and (C) increase in area of protrusions from spheroids grown in agarose and Matrigel from e1/control and e1/*Sox9*-KO cells. (*n* = 8, mean + S.D.) ** P=0.0013 in B and P=0.002 in C.

e1, embryonic mammary progenitor cell 1; Co, control, non-targeted cells; KO, knockout; *Sox9*-targeted cells.

Supplementary Figure 7

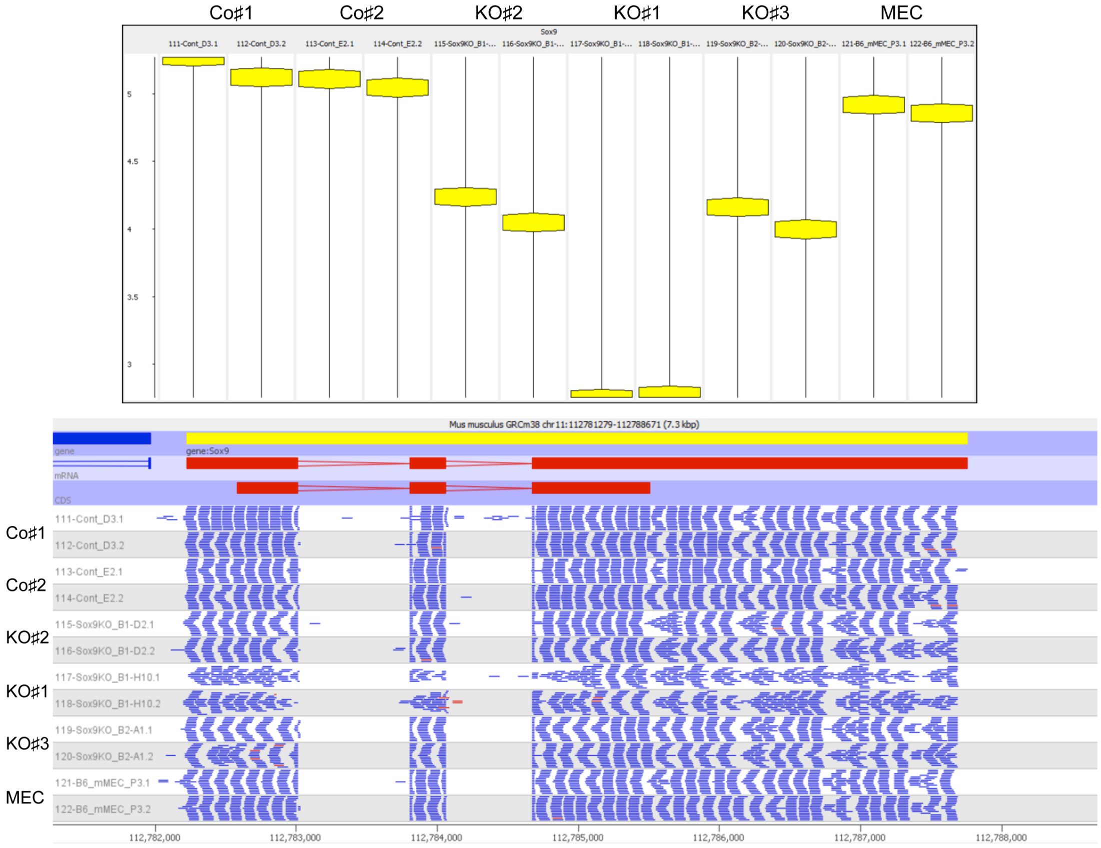

RNA-seq results of *Sox9* expression in e1/control versus e1/*Sox9*-KO cell lines.

Supplementary Figure 8

	Sox9+	Sox9-
**SC activity**	moderate	high
**Mammosphere morphology**	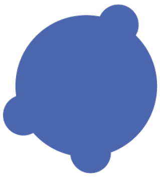	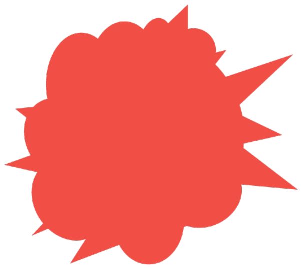
**Cell state**	M<br>Zeb1+	M/E<br>Zeb1-
**Lactational competence**	high	low

Sox9 regulation of embryonic mammary progenitor cells. Sox9 alters cell state and modulates stem cell activity, luminal progenitor function, and mammosphere morphology.
