# Supplementary material for: Sox9 regulates cell state and activity of embryonic mouse mammary progenitor cells
Source: Commun Biol. 2018 Dec 13;1:228. doi: 10.1038/s42003-018-0215-3 (PMC6292906; doi:10.1038/s42003-018-0215-3)
Supplement: Supplementary file 2 — Description of Additional Supplementary Files [file 42003_2018_215_MOESM2_ESM.docx]

**Description of Additional Supplementary Files**

**File Name**: Supplementary Data 1

**Description**: RNA sequencing data of embryonic mammary progenitor clones.

**File Name**: Supplementary Data 2

**Description**: RNA sequencing data of E12.5-stage embryonic mammary tissues.

**File Name**: Supplementary Data 3

**Description**: RNA sequencing data of eMPC.1 Sox9-Co and KO cells.

**File Name**: Supplementary Data 4

**Description**: Taqman probes, antibodies, and crRNA target sequences

**File Name**: Supplementary Data 5

**Description**: Source data underlying the graphs and charts presented in Figures 4B, 5B, 5C, 6B, 7D, 7F, 7G, 8D and Supplementary Figures 6B and 6C.
